# *In vitro* biologic efficacy of sunitinib drug-eluting beads on human colorectal and hepatocellular carcinoma—A pilot study

**DOI:** 10.1371/journal.pone.0174539

**Published:** 2017-04-06

**Authors:** Steven Lahti, Johannes M. Ludwig, Minzhi Xing, Lingyi Sun, Dexing Zeng, Hyun S. Kim

**Affiliations:** 1 Interventional Oncology Translational Laboratory, Pittsburgh School of Medicine, Presbyterian South Tower, Pittsburgh, PA, United States of America; 2 Division of Interventional Radiology, Department of Radiology and Biomedical Imaging, Yale School of Medicine, New Haven, CT, United States of America; 3 Molecular Imaging Laboratory, Department of Radiology, University of Pittsburgh School of Medicine, Pittsburgh, PA, United States of America; 4 Yale Cancer Center, Yale School of Medicine, New Haven, New Haven, CT, United States of America; Institute of Biochemistry and Biotechnology, TAIWAN

## Abstract

**Purpose:**

Sunitinib drug eluting beads (DEB) are a novel anti-angiogenic bead preparation for use in transarterial chemoembolization. However, systematic studies of sunitinib DEB’s effect on cancer cells have not been reported. Herein, we assess their direct biologic efficacy against carcinoma cell lines and correlate cell viability with drug release *in vitro*.

**Materials and methods:**

Sunitinib-HCl (10mg/mL) in Milli-Q water was mixed with LC Bead® 300–500μm (Biocompatibles UK Ltd.). Loading and release were assessed by measurement of drug UV absorbance using UV-visible spectrophotometer. Viability of human colorectal cancer (CRC, HCT116 and HT29) and hepatocellular carcinoma (HCC, HepG2) cells upon exposure to sunitinib DEB was measured using a bioluminescent assay. Drug concentration during exposure was quantified using HPLC.

**Results:**

When added to cultured HepG2 cells, sunitinib DEB rapidly inhibited viability with a significant decrease observed within 1 hour of incubation. Viability of HCT116 and HT29 cells decreased relatively slower, with significant reductions observed after 8 and 24 hours, respectively. After 24 hours there was nearly complete inhibition of all three cell lines. There was no difference in viability observed between cells treated with 5 μl, 10 μL, or 20 μL of sunitinib DEB. HPLC analysis of the cell culture supernatant demonstrated saturation of the cell medium within approximately 4 hours for each amount added, with sunitinib achieving a final concentration of 17.61 μM (SE ±1.01).

**Conclusions:**

Sunitinib can be efficiently loaded to and released from LC beads, and the resulting sunitinib DEB demonstrate strong *in vitro* inhibition of human CRC and HCC cells.

## Introduction

Transarterial chemoembolization (TACE) is a form of locoregional therapy that prolongs survival in patients with unresectable hepatocellular carcinoma (HCC), cholangiocarcinoma, and a variety of hepatic metastases such as colorectal carcinoma [1]. The development of drug-eluting beads (DEB) as combined embolic agents and drug-delivery vehicles for use in TACE has standardized treatment regimens, aiming at increasing intratumoral drug concentration and objective tumor response, while decreasing systemic toxicity compared to the conventional TACE based on the oily lipidol/drug emulsion [1,2]. However, the meta-analysis from Facciorusso et al. included 12 studies demonstrating equality between the two therapies in terms of tumor response, survival and severe adverse events in patients with non-resectable HCC [2]. Moreover, a recent randomized trial (~50 patients per cohort) compared bland transarterial embolization (TAE) vs. doxorubicin DEB-TACE demonstrating no survival differences in treatment response, progression free survival, overall survival or adverse events rate [3]. Although no comparable data on the efficacy of Irinotecan loaded DEB in the treatment of colorectal liver metastases are available [1,4], the data thus far compels further investigation of other anticancer drugs that may be loaded onto DEB and upon release into the tumor, exert a more potent antineoplastic effect than those already described.

One major limitation of DEB-TACE is its short-lived effect, which results in part because DEB induced hypoxia stimulates neoangiogenesis and a recovery of tumor blood supply. Numerous studies have documented increased expression of vascular endothelial growth factor (VEGF) in TACE treated tissues, as well as significantly increased serum/plasma levels of VEGF thereafter [5–7]. Through its effects on neoangiogenesis and vascular permeability, VEGF likely contributes to local recurrence and extrahepatic metastasis as has been investigated by previous studies [8–10].

Fuchs et al have recently synthesized an antiangiogenic DEB loaded with sunitinib and have characterized the *in vitro* release characteristics, biologic efficacy against human umbilical vein endothelial cells (HUVECs) and the *in vivo* release characteristics after TACE using a rabbit model [11]. In this study they demonstrated that beads loaded to 30 mg/g bead had a release half-life of 1.1 and 1.6 hrs for 70–150 and 100–300 μm beads, respectively. They also observed maximum *in vitro* release of 81 and 82% for these two bead sizes. Sunitinib DEB, furthermore, were shown to inhibit HUVEC proliferation and migration in response to VEGF stimulation. In a subsequent efficacy study [7], sunitinib loaded DEB achieved tumor growth arrest (-2%) within 14 days of monitoring compared to oral sunitinib treatment (+1853%) and unloaded DEB intra-arterial injection (+42%) (p = 0.044) of VX2 tumor implants in rabbits. Furthermore, a complete treatment response was observed in 60% of sunitinib loaded and in 43% of unloaded DEB 14 days after treatment start.

Sunitinib (SU011248), a small molecule inhibitor of class III and class IV receptor tyrosine kinases, including VEGFR1-3, PDGFR-α/β, c-Kit, Flt3, and RET, is currently approved for the treatment of renal cell carcinoma (RCC) and imatinib-refractory gastrointestinal stromal tumors (GIST) [12,13]. Sunitinib has potent antiangiogenic activity owing to its strong activity against VEGF and PDGF signaling [13], which is its primary mechanism of action in RCC at plasma concentrations achievable with oral delivery (50-100ng/ml, ~0.12–0.25 μM) [14]. However, it has been observed that at doses above 5 μM sunitinib can mediate a direct effect on carcinoma cells *in vitro* via activation of apoptosis [15]. Whether or not sunitinib DEB, as described by Fuchs et al can deliver a cytotoxic level of sunitinib has not been determined, but would be informative in designing further bead preparations. The adverse events associated with the systemic use of sunitinib, including diarrhea, fatigue, nausea, bone marrow suppression in the form of anemia and cytopenia, hypertension, hypothyroidism, and cardiac toxicity, may be expected to occur less frequently with DEB delivery [16].

In this study, we report the *in vitro* evaluation of the direct effects of sunitinib DEB against several human carcinoma cell lines and correlate cell viability during exposure with drug release.

## Materials and methods

This study was approved by the Animal Care and Use Committee of University of Pittsburgh, and all animal care and procedures were performed under institutional guidelines and have been performed in accordance with the ethical standards as laid down in the 1964 Declaration of Helsinki and its later amendments.

### Generation of sunitinib loaded DEB

Free base sunitinib was supplied in solid form (LC Labs, Woburn, MA). Loading was performed as previously described [11]. Briefly, sunitinib was converted to its hydrochloride salt by reaction with 1N hydrochloric acid, then mixed with Milli-Q water to achieve a stock solution of sunitinib-HCl at 10mg/ml. After pipetting up and down swiftly with wide bore pipette tip, 1ml LC Bead® 300–500μm (Biocompatibles UK Ltd.) was transferred to the barrel of a filtered syringe with the plunger removed. The plunger was then inserted into the syringe barrel to expel the bead storage solution. 1ml hydrated bead was washed with pure water prior to the addition of 0.5 or 1ml of sunitinib-HCl stock (10mg/ml). Loading was confirmed by UV-visible spectrophotometer as described below.

### Quantitative evaluation of sunitinib DEB loading

Using the filtered syringe, 10 μL of supernatant was expelled at 5, 10, 20, 30, and 60 minutes, and then at 2, 4, 6, and 24 hours. UV absorbance at 425 nm was measured with a multi-modal microplate reader (Synergy H4, BioTek Instruments Inc., Winooski, VT, USA), and the results were compared to reference standards to quantify the proportion of drug complexed to beads at each time point.

### Cell culture

HCT116 (CCL-247™) and HT29 (HTB-38™) human colorectal and HepG2 (HB-8065™) human hepatocellular carcinoma cell lines were obtained from the American Type Culture Collection (ATCC, Manassas, VA, USA). The HCT116 and HT29 cells were maintained in McCoy’s 5A medium +2.2 g/L Sodium Bicarbonate (HyClone Laboratories, Logan, UT, USA). HepG2 cells were maintained in Eagle’s minimum essential medium (EMEM). Both cell culture media were supplemented with 2 mM L-Glutamine (Lonza, Walkersville, MD, USA), 10% fetal calf serum (FCS) (HyClone Laboratories, Logan, UT, USA) and Penicillin/Streptomycin; 100 μg/ml each (Gibco, Grand Island, NY, USA). All cells were kept in a humidified atmosphere at 5% CO2 and 37°C.

### Cell viability assay

Cells were plated in a 96 well clear bottom culture plate at a density of 50,000 cells/well in triplicate. Cells were allowed to incubate in 200 μl culture medium for 48 hours prior to bead addition. Cells were then treated with 5 μl of unloaded or sunitinib loaded (5 mg/ml) DEB, or an equal volume of culture medium. Prior to each assay, the beads and supernatant were removed using a wide bore pipette tip under vacuum and replaced with a 1:1 mixture of medium and CellTiter-Glo® (Promega Inc., Madison, WI, USA) cell viability assay reagent. The cell/reagent mixture was allowed to incubate for 10 minutes at room temperature. Bioluminescence was measured according to manufacturer’s instructions using a microplate reader. The average luminescence was calculated for each triplicate and the viability expressed as a percentage of that of time-matched unexposed controls.

### Quantitative evaluation of sunitinib release

5 μL of supernatant was sampled from sunitinib DEB treated HCT116 cells at 0.25, 0.5, 1, 2, and 24 hours of exposure, and it was then diluted 1:10 in phosphate buffered saline prior to high performance liquid chromatography (HPLC) analysis. HPLC analysis was performed using a c18 Luna (150 * 4.6 mm) column (Phenomenex, Torrance, CA, USA) attached to a Waters 1525 binary pump (Waters Corporation, Milford, MA, USA) with a mixed solvent of water (20%) and acetonitrile (80%) containing 0.1% TFA, and UV detector set to 425nm. Sunitinib concentration was calculated based on a standard curve of 20 μM sunitinib in cell culture medium. To evaluate the maximal release fraction of sunitinib from DEB after 24h, loaded DEB (5 mg/ml) were incubated in a surplus of supplemented McCoy’s 5a cell culture medium so that the maximal achievable concentration could be 20 μM in case of complete eluting from DEB.

### Statistical analysis

All data is represented as mean ± standard error (SE) if not stated otherwise. Groups were compared using the 2-tailed Student t-test for 2 groups or the One-Way ANOVA including Bonferroni Post-Hoc test for three or more groups. Loading and eluting curves were calculated using a hyperbola non-linear regression curve fitting model of loading vs. time. Statistical differences between between eluting curves were determined using the Extra sum-of-squares F test. All values are expressed as mean with standard error (SE) unless stated otherwise All calculations were performed with PRISM 6 (GraphPad Software Inc., La Jolla, CA, USA). P-values <0.05 were considered statistically significant.

## Results

### Sunitinib DEB loading and eluting kinetics

**Fig 1:**
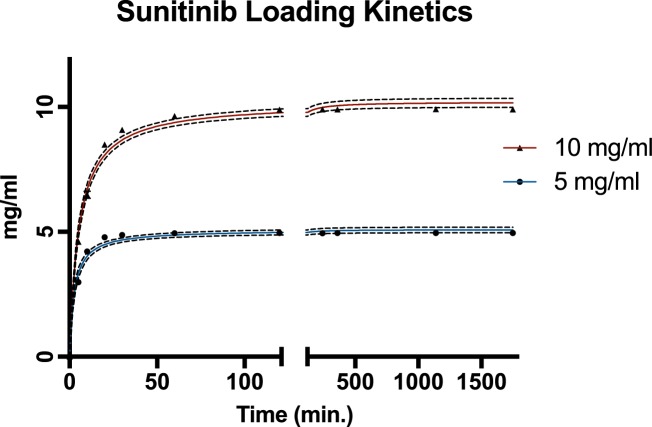
Sunitinib loading onto drug-eluting bead (DEB) is quick and complete. **Sunitinib** loading kinetics of 5 mg/ml (blue line) and 10 mg/ml (red line) DEB are plotted achieving >99% loading efficacy. n = 2 replicates. Error bars display standard errors of data points. Dashed line displays 95% CI for the fitted curves.

Sunitinib loading onto drug-eluting bead was demonstrated to occur rapidly and efficiently (S1 Table). For 5 and 10 mg/ml hydrated DEB, a maximal loading of > 99% was achieved within 60 and 120 minutes, respectively. Loading of 50% onto DEB occurred within 2.6 min. (SE ±0.39) and 5.2 min (SE ±0.44) for 5 and 10 mg/ml respectively (Fig 1).

**Fig 2:**
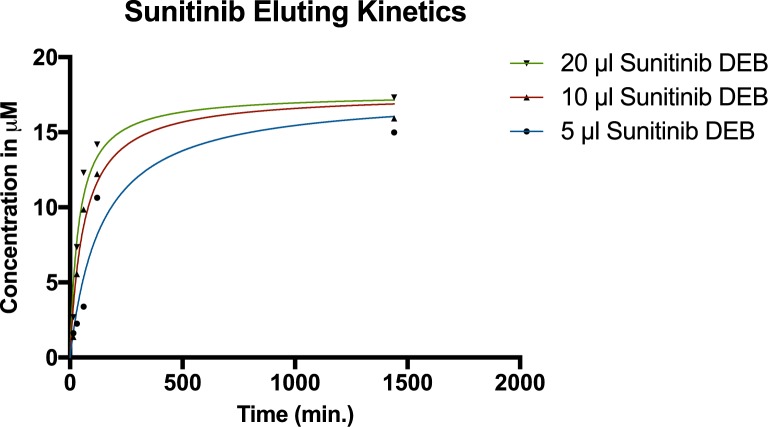
Sunitinib eluting kinetics. Various amounts of sunitinib loaded DEB (5, 10 or 20 μl; 5 mg/ml) were incubated in complete cell culture media at 37°C/5%CO_2_. n = 1.

The analysis of drug release in the cell culture medium showed the release to be concentration dependent and saturating (S2 Table). HPLC analysis of the cell medium demonstrated drug saturation after approximately 180 minutes irrespective of the volume of bead added. The drug concentration in the cell culture medium after incubation for 1440 minutes with 5 mg/ml sunitinib DEBs was calculated to be 16.76 μM (SE ±2.45), 17.1 μM (SE ±1.62), and 18.54 μM (SE: ±1.62) for 5, 10 and 20 μL DEB respectively without a statistical difference between groups (p = 0.76) (Fig 2). Due to the similarity of data sets, an overall estimated achievable elution concentration of 17.61 μM (SE ±1.01) as saturation point could be calculated. The half-maximal elution time of total achieved concentrations for 5, 10 and 20 μl of loaded DEB was 127 min. (SE ±53.41), 57.8 min (SE ±16.9), and 43.2 min. (SE ±12.26) (p = 0.01). The maximal average eluted percentage of sunitinib from the DEB was 5.36% (SE: ±0.8%), 2.72% (SE: ±0.26%), and 1.48% (SE: ±0.13%) for the elution of 5, 10, and 20 μl of loaded DEB respectively. Since the eluted fraction was lower than expected, we tested the eluting fraction in greater medium volumes. After 24h, the eluted fraction was measured to be 49.64% (SE: ±1.3%) achieving a final concentration of 9.93 μM (SE: ±0.26 μM) which indicates that the eluted percentage of sunitinib from DEB during the cell treatment was most likely limited by cell culture medium solubility.

### Biologic efficacy of sunitinib loaded DEB

**Fig 3:**
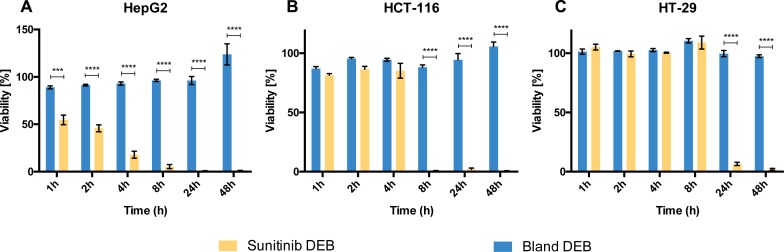
Sunitinib loaded DEB exert a direct toxic effect on cancer cells. Viability assessment of HepG2 (A) HCT-116 (B), and HT-29 (C) cell lines after treatment with 1h to 48h with bland DEB (blue bars) or 5 μl of 5 mg/ml sunitinib loaded DEB (yellow bars) was performed. Significance levels: *** (p<0.001), **** (p<0.0001). Mean values with SE are plotted; n = 3–8. Viability is expressed as a percentage of that of time matched untreated controls and thus calculated values may be greater than 100%. For raw data see S3 Table.

In HepG2 cells, sunitinib loaded beads (5 μl; 5 mg/ml) demonstrated strong and rapid inhibition based on the cell viability shown in Fig 3A. There was a significant decrease in viability relative to cells treated with unloaded DEBs within 1 hour of exposure. By 4 hours sunitinib DEB treated HepG2 cell viability was less than half that of untreated cells. By 24 hours there was nearly complete inhibition of HepG2 cell viability. The relative viability of cells treated with unloaded DEB remained close to 100% at all time points, suggesting that the unloaded beads themselves had no significant effect on cell viability.

In contrast, the two CRC cell lines had a relatively slower response to sunitinib DEB. Significant differences in viability relative to cells treated with unloaded DEB were not observed until 8 hours of exposure for HCT116 (Fig 3B) and 24 hours of exposure for HT29 cells (Fig 3C). However, the response to sunitinib DEB was essentially complete after 24 hours for both of these cell lines (95% confidence interval includes viability of 0).

## Discussion

The results of this study demonstrate the potential direct antitumor effects of sunitinib DEB. Sunitinib was efficiently loaded to DEB and quickly released in cell culture media. After 24 hours of incubation, sunitinib achieved a final treatment concentration of ~16–18 μM under the conditions used in this assay. We believe that, with a total eluting percentage of ~1.5–5% of originally loaded sunitinib on DEB, solubility in the culture media is the limiting factor to drug release during the *in vitro* assay as the release profile demonstrated a plateau effect after 24 hours. Since the eluted fraction was lower than expected, we tested the eluting fraction in greater medium volumes to avoid medium saturation and demonstrated an eluting fraction of ~50% within 24h. Importantly, despite only minimal drug release fraction from beads during the sunitinib DEB treatment of cancer cells, the achieved sunitinib concentration in the medium were sufficient to significantly reduce the viability of all tested HCC and CRC cell lines.

In previous studies, Fuchs et al have reported on the biologic efficacy of sunitinib DEB against HUVEC proliferation and migration, however, they only observed cytostatic effects upon exposure of carcinoma cells. In this study we show that at doses achievable with DEBs *in vitro* sunitinib can have a direct effect on tumor cell viability for both, HCC and CRC cell lines. The difference in these results may relate to differences in the number of cells used during the assays, the length of exposure, or the cell lines themselves. Indeed, our results demonstrate that under the same experimental conditions cell lines respond differentially to sunitinib DEB exposure. The HepG2 HCC cell line, for instance, responded much faster than the two CRC cell lines (HCT116 and HT29). This may relate to differences between these cell lines in receptor expression and/or function. VEGFR-1 mRNA transcription is upregulated and leads to increased VEGFR-1 expression on the membrane of HCC cells, in addition to stromal cells, in HCC tissues [17]. There is emerging evidence that autocrine VEGF signaling exists and promotes HCC cell proliferation and viability in an angiogenesis-independent manner [18]. On the other hand, while VEGFR-1 expression has also been observed in CRC cell lines, including HT29 [19], it appears to play a larger role in epithelial-mesenchymal transition than it does in cell proliferation [20,21].

The mechanism of sunitinib’s direct antitumor effect in CRC is not completely understood. *In vitro* studies involving multiple cancer cell lines suggests a possible pro-apoptotic effect of sunitinib via its induction of BH3-only proteins. Dose dependent upregulation of p53-upregulated modulator of apoptosis (PUMA) has been observed in both wild type and *p53* knockout cell lines, and is necessary for sunitinib’s pro-apoptotic activity in CRC cells [15]. The clinical significance of this direct antitumor effect at doses achievable with oral dosing regimens is debatable. Moreover, due to its poor water solubility, protein binding, and accumulation in liver tissue, it is difficult to compare sunitinib malate—the commercial formulation—with the hydrochloride salt used in DEB [22]. Nevertheless, Fuchs et al. demonstrated that sunitinib (2 mg/kg) tumor delivery via DEB increased the intratumoral concentration up to 3.5x after 6h and 1.2x after 24h compared to oral administration in a rabbit liver cancer model. Importantly, peak plasma drug levels achieved in their study did not reach the therapeutic threshold of 50 ng/mL so increased dosages should be tolerable. We believe this pilot study provides sufficient evidence to warrant further *in vivo* studies of the beads potential direct efficacy alone and in combination with cytotoxic DEB.

## Conclusion

In conclusion, sunitinib can be efficiently loaded to and released from DEB and strongly inhibits viability of human colorectal cancer and hepatocellular carcinoma cells *in vitro*. The direct antitumor effect of the beads, in combination with their putative antiangiogenic properties, makes them an attractive bead preparation candidate for use in DEB-TACE treatment of HCC and CRC liver lesions warranting further investigation.

## Supporting information

S1 Table Sunitinib loading data. Measurement data represent the amount of compound loaded onto drug-eluting beads in mg. Both amounts (5 and 10 mg) get almost completely loaded.
(DOCX)

S2 Table Sunitinib elution data. Measurement data represent the concentration of free sunitinib released from the beads during incubation in the cell culture medium over time. Regardless of the amount of bead added to wells the concentration plateaus around 15–17 μM.
(DOCX)

S3 Table Cell viability data. Measurement data represent the calculated viability (fluorescence signal in bead treated sample / mean fluorescence signal in 3 untreated samples) for HCT116, HT29, and HepG2 cells exposed to bland and sunitinib DEB over time. Mean and standard deviation (SD) are provided in the far right columns.
(DOCX)

S1 Data Raw data. (DOCX)

## References

[1] Habib A, Desai K, Hickey R, Thornburg B, Lewandowski R, Salem R (2015) Transarterial approaches to primary and secondary hepatic malignancies. Nat Rev Clin Oncol 12: 481–489. doi: 10.1038/nrclinonc.2015.78 25985939 10.1038/nrclinonc.2015.78

[2] Facciorusso A, Di Maso M, Muscatiello N (2016) Drug-eluting beads versus conventional chemoembolization for the treatment of unresectable hepatocellular carcinoma: A meta-analysis. Dig Liver Dis. 10.1016/j.dld.2016.02.005 26965785

[3] Brown KT, Do RK, Gonen M, Covey AM, Getrajdman GI, Sofocleous CT, et al (2016) Randomized Trial of Hepatic Artery Embolization for Hepatocellular Carcinoma Using Doxorubicin-Eluting Microspheres Compared With Embolization With Microspheres Alone. J Clin Oncol. 10.1200/JCO.2015.64.0821 PMC4966514 26834067

[4] Liu DM, Thakor AS, Baerlocher M, Alshammari MT, Lim H, Kos S, et al (2015) A review of conventional and drug-eluting chemoembolization in the treatment of colorectal liver metastases: principles and proof. Future Oncol 11: 1421–1428. doi: 10.2217/fon.15.3 25602287 10.2217/fon.15.3

[5] Gadaleta CD, Ranieri G (2011) Trans-arterial chemoembolization as a therapy for liver tumours: New clinical developments and suggestions for combination with angiogenesis inhibitors. Crit Rev Oncol Hematol 80: 40–53. doi: 10.1016/j.critrevonc.2010.10.005 21067940 10.1016/j.critrevonc.2010.10.005

[6] Li X, Feng GS, Zheng CS, Zhuo CK, Liu X (2003) Influence of transarterial chemoembolization on angiogenesis and expression of vascular endothelial growth factor and basic fibroblast growth factor in rat with Walker-256 transplanted hepatoma: an experimental study. World J Gastroenterol 9: 2445–2449. doi: 10.3748/wjg.v9.i11.2445 14606073 10.3748/wjg.v9.i11.2445 PMC4656518

[7] Bize P, Duran R, Fuchs K, Dormond O, Namur J, Decosterd LA, et al (2016) Antitumoral Effect of Sunitinib-eluting Beads in the Rabbit VX2 Tumor Model. Radiology: 150361. 10.1148/radiol.2016150361 26919561

[8] Shim JH, Park JW, Kim JH, An M, Kong SY, Nam BH, et al (2008) Association between increment of serum VEGF level and prognosis after transcatheter arterial chemoembolization in hepatocellular carcinoma patients. Cancer Sci 99: 2037–2044. doi: 10.1111/j.1349-7006.2008.00909.x 19016764 10.1111/j.1349-7006.2008.00909.x PMC11158304

[9] Sergio A, Cristofori C, Cardin R, Pivetta G, Ragazzi R, Baldan A, et al (2008) Transcatheter arterial chemoembolization (TACE) in hepatocellular carcinoma (HCC): the role of angiogenesis and invasiveness. Am J Gastroenterol 103: 914–921. doi: 10.1111/j.1572-0241.2007.01712.x 18177453 10.1111/j.1572-0241.2007.01712.x

[10] Li X, Feng GS, Zheng CS, Zhuo CK, Liu X (2004) Expression of plasma vascular endothelial growth factor in patients with hepatocellular carcinoma and effect of transcatheter arterial chemoembolization therapy on plasma vascular endothelial growth factor level. World J Gastroenterol 10: 2878–2882. doi: 10.3748/wjg.v10.i19.2878 15334691 10.3748/wjg.v10.i19.2878 PMC4572123

[11] Fuchs K, Bize PE, Dormond O, Denys A, Doelker E, Borchard G, et al (2014) Drug-eluting beads loaded with antiangiogenic agents for chemoembolization: in vitro sunitinib loading and release and in vivo pharmacokinetics in an animal model. J Vasc Interv Radiol 25: 379–387, 387 e371-372. doi: 10.1016/j.jvir.2013.11.039 24468044 10.1016/j.jvir.2013.11.039

[12] Mendel DB, Laird AD, Xin X, Louie SG, Christensen JG, Li G, et al (2003) In vivo antitumor activity of SU11248, a novel tyrosine kinase inhibitor targeting vascular endothelial growth factor and platelet-derived growth factor receptors: determination of a pharmacokinetic/pharmacodynamic relationship. Clin Cancer Res 9: 327–337. 12538485

[13] Faivre S, Demetri G, Sargent W, Raymond E (2007) Molecular basis for sunitinib efficacy and future clinical development. Nat Rev Drug Discov 6: 734–745. doi: 10.1038/nrd2380 17690708 10.1038/nrd2380

[14] Huang D, Ding Y, Li Y, Luo WM, Zhang ZF, Snider J, et al (2010) Sunitinib acts primarily on tumor endothelium rather than tumor cells to inhibit the growth of renal cell carcinoma. Cancer Res 70: 1053–1062. doi: 10.1158/0008-5472.CAN-09-3722 20103629 10.1158/0008-5472.CAN-09-3722

[15] Sun J, Sun Q, Brown MF, Dudgeon C, Chandler J, Xu X, et al (2012) The multi-targeted kinase inhibitor sunitinib induces apoptosis in colon cancer cells via PUMA. PLoS One 7: e43158 doi: 10.1371/journal.pone.0043158 22912816 10.1371/journal.pone.0043158 PMC3422222

[16] Schmid TA, Gore ME (2016) Sunitinib in the treatment of metastatic renal cell carcinoma. Ther Adv Urol 8: 348–371. doi: 10.1177/1756287216663979 27904651 10.1177/1756287216663979 PMC5117167

[17] Li T, Zhu Y, Qin CY, Yang Z, Fang A, Xu S, et al (2012) Expression and prognostic significance of vascular endothelial growth factor receptor 1 in hepatocellular carcinoma. J Clin Pathol 65: 808–814. doi: 10.1136/jclinpath-2012-200721 22734007 10.1136/jclinpath-2012-200721

[18] Peng S, Wang Y, Peng H, Chen D, Shen S, Peng B, et al (2014) Autocrine vascular endothelial growth factor signaling promotes cell proliferation and modulates sorafenib treatment efficacy in hepatocellular carcinoma. Hepatology 60: 1264–1277. doi: 10.1002/hep.27236 24849467 10.1002/hep.27236

[19] Fan F, Wey JS, McCarty MF, Belcheva A, Liu W, Bauer TW, et al (2005) Expression and function of vascular endothelial growth factor receptor-1 on human colorectal cancer cells. Oncogene 24: 2647–2653. doi: 10.1038/sj.onc.1208246 15735759 10.1038/sj.onc.1208246

[20] Bates RC, Goldsmith JD, Bachelder RE, Brown C, Shibuya M, Oettgen P, et al (2003) Flt-1-dependent survival characterizes the epithelial-mesenchymal transition of colonic organoids. Curr Biol 13: 1721–1727. 14521839 10.1016/j.cub.2003.09.002

[21] Goel HL, Mercurio AM (2013) VEGF targets the tumour cell. Nat Rev Cancer 13: 871–882. doi: 10.1038/nrc3627 24263190 10.1038/nrc3627 PMC4011842

[22] (2014) Sutent Product Monograph.

