# Supplementary material for: In vitro biologic efficacy of sunitinib drug-eluting beads on human colorectal and hepatocellular carcinoma—A pilot study
Source: PLoS One. 2017 Apr 6;12(4):e0174539. doi: 10.1371/journal.pone.0174539 (PMC5383050; doi:10.1371/journal.pone.0174539)
Supplement: S1 Table — Measurement data represent the amount of compound loaded onto drug-eluting beads in mg. Both amounts (5 and 10 mg) get almost completely loaded. (DOCX) [file pone.0174539.s001.docx]

| **Time (Minutes)** | **Loaded amount: 5 mg** | | **Loaded amount: 10 mg** | |
| --- | --- | --- | --- | --- |
|  | **Measurement 1** | **Measurement 2** | **Measurement 1** | **Measurement 2** |
| 0 | 0 | 0 | 0 | 0 |
| 5 | 2.949952 | 3.015776 | 4.534056 | 4.6672 |
| 10 | 4.21856 | 4.215568 | 6.338232 | 6.622472 |
| 20 | 4.781056 | 4.781804 | 8.477512 | 8.505936 |
| 30 | 4.871564 | 4.870068 | 9.078904 | 9.086384 |
| 60 | 4.947112 | 4.945616 | 9.644392 | 9.632424 |
| 120 | 4.95534 | 4.953096 | 9.898712 | 9.897216 |
| 240 | 4.953096 | 4.92916 | 9.9032 | 9.913672 |
| 360 | 4.95534 | 4.954592 | 9.904696 | 9.904696 |
| 1140 | 4.956088 | 4.954592 | 9.91068 | 9.91068 |
| 1750 | 4.954966 | 4.954218 | 9.91068 | 9.909184 |

**S1 Table. Sunitinib loading data.** Measurement data represent the amount of compound loaded onto drug-eluting beads in mg. Both amounts (5 and 10 mg) get almost completely loaded.
