# Supplementary material for: In vitro biologic efficacy of sunitinib drug-eluting beads on human colorectal and hepatocellular carcinoma—A pilot study
Source: PLoS One. 2017 Apr 6;12(4):e0174539. doi: 10.1371/journal.pone.0174539 (PMC5383050; doi:10.1371/journal.pone.0174539)
Supplement: S2 Table — Measurement data represent the concentration of free sunitinib released from the beads during incubation in the cell culture medium over time. Regardless of the amount of bead added to wells the concentration plateaus around 15–17 μM. (DOCX) [file pone.0174539.s002.docx]

| **Time (min)** | **5 μl Sunitinib DEB** | **10 μl Sunitinib DEB** | **20 μl Sunitinib DEB** |
| --- | --- | --- | --- |
| 0 | 0 | 0 | 0 |
| 15 | 1.617627 μM | 1.371074 μM | 2.69404 μM |
| 30 | 2.25 μM | 5.55 μM | 7.366516 μM |
| 60 | 3.397617 μM | 9.86 μM | 12.30359 μM |
| 120 | 10.63785 μM | 12.22 μM | 14.19783 μM |
| 1440 | 14.989 μM | 15.91769 μM | 17.33086 μM |

**S2 Table. Sunitinib elution data.** Measurement data represent the concentration of free sunitinib released from the beads during incubation in the cell culture medium over time. Regardless of the amount of bead added to wells the concentration plateaus around 15-17 µM.
