# Supplementary material for: In vitro biologic efficacy of sunitinib drug-eluting beads on human colorectal and hepatocellular carcinoma—A pilot study
Source: PLoS One. 2017 Apr 6;12(4):e0174539. doi: 10.1371/journal.pone.0174539 (PMC5383050; doi:10.1371/journal.pone.0174539)
Supplement: S3 Table — Measurement data represent the calculated viability (fluorescence signal in bead treated sample / mean fluorescence signal in 3 untreated samples) for HCT116, HT29, and HepG2 cells exposed to bland and sunitinib DEB over time. Mean and standard deviation (SD) are provided in the far right columns. (DOCX) [file pone.0174539.s003.docx]

| **HCT116 drug** | | | | | | | | | | |
| --- | --- | --- | --- | --- | --- | --- | --- | --- | --- | --- |
| **Time (hr)** | **Trial 1** | **Trial 2** | **Trial 3** | **Trial 4** | **Trial 5** | **Trial 6** | **Trial 7** | **Trial 8** | **Mean** | **SD** |
| 1 | 0.80548 | 0.85765 | 0.83157 | 0.79047 | 0.79119 |  |  |  | 0.81527 | 0.02895 |
| 2 | 0.90917 | 0.86489 | 0.86534 | 0.90827 | 0.78581 |  |  |  | 0.86670 | 0.05020 |
| 4 | 0.95134 | 0.70064 | 0.79813 | 0.95775 |  |  |  |  | 0.85196 | 0.12498 |
| 8 | 0.00890 | 0.00843 | 0.00954 | 0.00636 | 0.00970 |  |  |  | 0.00858 | 0.00134 |
| 24 | 0.00740 | 0.00363 | 0.00334 | 0.00363 | 0.06743 | 0.02491 | 0.03629 |  | 0.02094 | 0.02417 |
| 48 | 0.00462 | 0.00346 | 0.00365 | 0.00385 | 0.02442 | 0.00265 | 0.00221 | 0.00232 | 0.00590 | 0.00753 |
| **HCT116 bland bead** | | | | | | | | | | |
| **Time (hr)** | **Trial 1** | **Trial 2** | **Trial 3** | **Trial 4** | **Trial 5** | **Trial 6** | **Trial 7** | **Trial 8** | **Mean** | **SD** |
| 1 | 0.89482 | 0.87874 | 0.83943 |  |  |  |  |  | 0.87099 | 0.02850 |
| 2 | 0.96249 | 0.96656 | 0.93719 |  |  |  |  |  | 0.95541 | 0.01591 |
| 4 | 0.93737 | 0.92964 | 0.96975 |  |  |  |  |  | 0.94559 | 0.02128 |
| 8 | 0.85985 | 0.92248 | 0.86207 |  |  |  |  |  | 0.88146 | 0.03554 |
| 24 | 0.80357 | 0.90846 | 0.78979 | 1.08975 | 1.05480 | 1.02036 |  |  | 0.94445 | 0.12970 |
| 48 | 1.14839 | 1.09070 | 1.14935 | 1.05009 | 0.91735 | 0.98118 |  |  | 1.05618 | 0.09307 |
| **HT29 drug** | | | | | | | | | | |
| **Time (hr)** | **Trial 1** | **Trial 2** | **Trial 3** | **Trial 4** | **Trial 5** | **Trial 6** | **Trial 7** | **Trial 8** | **Mean** | **SD** |
| 1 | 1.05178 | 1.07316 | 1.12827 | 1.02898 | 0.97910 |  |  |  | 1.05226 | 0.05502 |
| 2 | 1.01092 | 1.03183 | 1.01206 | 1.01629 | 0.89678 |  |  |  | 0.99358 | 0.05475 |
| 4 | 0.99774 | 0.99635 | 1.00232 | 1.01328 | 1.01289 |  |  |  | 1.00452 | 0.00813 |
| 8 | 1.11928 | 1.11980 | 1.18710 | 0.93416 |  |  |  |  | 1.09008 | 0.10872 |
| 24 | 0.05149 | 0.03599 | 0.05164 | 0.02911 | 0.05665 | 0.11679 | 0.11769 |  | 0.06562 | 0.03655 |
| 48 | 0.00558 | 0.00634 | 0.00483 | 0.00483 | 0.00558 | 0.04115 | 0.01852 | 0.06789 | 0.01934 | 0.02331 |
| **HT29 bland bead** | | | | | | | | | | |
| **Time (hr)** | **Trial 1** | **Trial 2** | **Trial 3** | **Trial 4** | **Trial 5** | **Trial 6** | **Trial 7** | **Trial 8** | **Mean** | **SD** |
| 1 | 0.98242 | 1.00285 | 1.05606 |  |  |  |  |  | 1.01378 | 0.03801 |
| 2 | 1.02166 | 1.01545 | 1.01997 |  |  |  |  |  | 1.01902 | 0.00321 |
| 4 | 1.03779 | 1.00053 | 1.04158 |  |  |  |  |  | 1.02664 | 0.02269 |
| 8 | 1.13186 | 1.06946 | 1.11316 |  |  |  |  |  | 1.10482 | 0.03203 |
| 24 | 1.02207 | 0.91174 | 1.06823 | 0.97049 | 1.01163 |  |  |  | 0.99683 | 0.05893 |
| 48 | 0.95443 | 0.98551 | 0.96937 | 0.95636 | 1.02866 | 0.95489 |  |  | 0.97487 | 0.02897 |
| **HepG2 drug** | | | | | | | | | | |
| **Time (hr)** | **Trial 1** | **Trial 2** | **Trial 3** | **Trial 4** | **Trial 5** | **Trial 6** | **Trial 7** | **Trial 8** | **Mean** | **SD** |
| 1 | 0.42431 | 0.52127 | 0.60663 | 0.71022 | 0.47072 |  |  |  | 0.54663 | 0.11372 |
| 2 | 0.35198 | 0.52773 | 0.39098 | 0.53415 | 0.48478 |  |  |  | 0.45792 | 0.08232 |
| 4 | 0.10880 | 0.17971 | 0.08329 | 0.24912 | 0.27126 |  |  |  | 0.17844 | 0.08294 |
| 8 | 0.00908 | 0.02598 | 0.01972 | 0.09484 | 0.11674 |  |  |  | 0.05327 | 0.04894 |
| 24 | 0.01008 | 0.00964 | 0.01008 | 0.01052 | 0.01096 | 0.00958 | 0.00919 | 0.00919 | 0.00991 | 0.00063 |
| 48 | 0.01436 | 0.01299 | 0.01573 | 0.01299 | 0.01368 | 0.00601 | 0.00742 | 0.00813 | 0.01141 | 0.00365 |
| **HepG2 bland bead** | | | | | | | | | | |
| **Time (hr)** | **Trial 1** | **Trial 2** | **Trial 3** | **Trial 4** | **Trial 5** | **Trial 6** | **Trial 7** | **Trial 8** | **Mean** | **SD** |
| 1 | 0.91823 | 0.86602 | 0.87928 |  |  |  |  |  | 0.88785 | 0.02714 |
| 2 | 0.92858 | 0.90193 | 0.91328 |  |  |  |  |  | 0.91460 | 0.01338 |
| 4 | 0.93422 | 0.96348 | 0.89557 |  |  |  |  |  | 0.93109 | 0.03406 |
| 8 | 0.96620 | 0.94085 | 0.98153 |  |  |  |  |  | 0.96286 | 0.02055 |
| 24 | 1.02981 | 0.94082 | 1.04910 | 1.06270 | 0.80411 | 0.89414 |  |  | 0.96345 | 0.10227 |
| 48 | 1.32528 | 1.33485 | 1.20903 | 1.52244 | 0.71528 | 1.31712 |  |  | 1.23733 | 0.27504 |

**S3 Table. Cell viability data.** Measurement data represent the calculated viability (fluorescence signal in bead treated sample / mean fluorescence signal in 3 untreated samples) for HCT116, HT29, and HepG2 cells exposed to bland and sunitinib DEB over time. Mean and standard deviation (SD) are provided in the far right columns.
