# Supplementary material for: In vitro biologic efficacy of sunitinib drug-eluting beads on human colorectal and hepatocellular carcinoma—A pilot study
Source: PLoS One. 2017 Apr 6;12(4):e0174539. doi: 10.1371/journal.pone.0174539 (PMC5383050; doi:10.1371/journal.pone.0174539)
Supplement: S1 Data — (DOCX) [file pone.0174539.s004.docx]

**Supporting information for “In vitro Biologic Efficacy of Sunitinib Drug-Eluting Beads on Human Colorectal and Hepatocellular Carcinoma - a Pilot Study”**

Data for Figure 1 - Sunitinib Loading Curve:

| **Time (Minutes)** | **Loaded amount: 5 mg** | | **Loaded amount: 10 mg** | |
| --- | --- | --- | --- | --- |
|  | **Measurement 1** | **Measurement 2** | **Measurement 1** | **Measurement 2** |
| 0 | 0 | 0 | 0 | 0 |
| 5 | 2.949952 | 3.015776 | 4.534056 | 4.6672 |
| 10 | 4.21856 | 4.215568 | 6.338232 | 6.622472 |
| 20 | 4.781056 | 4.781804 | 8.477512 | 8.505936 |
| 30 | 4.871564 | 4.870068 | 9.078904 | 9.086384 |
| 60 | 4.947112 | 4.945616 | 9.644392 | 9.632424 |
| 120 | 4.95534 | 4.953096 | 9.898712 | 9.897216 |
| 240 | 4.953096 | 4.92916 | 9.9032 | 9.913672 |
| 360 | 4.95534 | 4.954592 | 9.904696 | 9.904696 |
| 1140 | 4.956088 | 4.954592 | 9.91068 | 9.91068 |
| 1750 | 4.954966 | 4.954218 | 9.91068 | 9.909184 |

Measurement data represent the amount of compound loaded onto drug-eluting beads in mg. Both amounts (5 and 10 mg) get almost completely loaded.

Data for Figure 2 – Sunitinib eluting:

| **Time (min)** | **5 μl Sunitinib DEB** | **10 μl Sunitinib DEB** | **20 μl Sunitinib DEB** |
| --- | --- | --- | --- |
| 0 | 0 | 0 | 0 |
| 15 | 1.617627 μM | 1.371074 μM | 2.69404 μM |
| 30 | 2.25 μM | 5.55 μM | 7.366516 μM |
| 60 | 3.397617 μM | 9.86 μM | 12.30359 μM |
| 120 | 10.63785 μM | 12.22 μM | 14.19783 μM |
| 1440 | 14.989 μM | 15.91769 μM | 17.33086 μM |

Measurement data represent the concentration of measured free sunitinib released from the beads during incubation in the cell culture medium over time. Regardless of the amount of bead added to wells the concentration plateaus around 15-17 μM.

Data for Figure 3 – Cell viability:

| **HCT116 drug** | | | | | | | | |
| --- | --- | --- | --- | --- | --- | --- | --- | --- |
| **Time (hr)** | **Trial 1** | **Trial 2** | **Trial 3** | **Trial 4** | **Trial 5** | **Trial 6** | **Trial 7** | **Trial 8** |
| 1 | 0.80548 | 0.85765 | 0.83157 | 0.79047 |  |  |  |  |
| 2 | 0.90917 | 0.86489 | 0.86534 | 0.90827 |  |  |  |  |
| 4 | 0.95134 | 0.70064 | 0.79813 | 0.95775 |  |  |  |  |
| 8 | 0.00890 | 0.00843 | 0.00954 | 0.00636 |  |  |  |  |
| 24 | 0.00740 | 0.00363 | 0.00334 | 0.00363 | 0.06743 | 0.02491 | 0.03629 |  |
| 48 | 0.00462 | 0.00346 | 0.00365 | 0.00385 | 0.02442 | 0.00265 | 0.00221 | 0.00232 |
| **HCT116 bland bead** | | | | | | | | |
| **Time (hr)** | **Trial 1** | **Trial 2** | **Trial 3** | **Trial 4** | **Trial 5** | **Trial 6** | **Trial 7** | **Trial 8** |
| 1 | 0.89482 | 0.87874 | 0.83943 |  |  |  |  |  |
| 2 | 0.96249 | 0.96656 | 0.93719 |  |  |  |  |  |
| 4 | 0.93737 | 0.92964 | 0.96975 |  |  |  |  |  |
| 8 | 0.85985 | 0.92248 | 0.86207 |  |  |  |  |  |
| 24 | 0.80357 | 0.90846 | 0.78979 | 1.08975 | 1.05480 | 1.02036 |  |  |
| 48 | 1.14839 | 1.09070 | 1.14935 | 1.05009 | 0.91735 | 0.98118 |  |  |
| **HT29 drug** | | | | | | | | |
| **Time (hr)** | **Trial 1** | **Trial 2** | **Trial 3** | **Trial 4** | **Trial 5** | **Trial 6** | **Trial 7** | **Trial 8** |
| 1 | 1.05178 | 1.07316 | 1.12827 | 1.02898 | 0.97910 |  |  |  |
| 2 | 1.01092 | 1.03183 | 1.01206 | 1.01629 | 0.89678 |  |  |  |
| 4 | 0.99774 | 0.99635 | 1.00232 | 1.01328 | 1.01289 |  |  |  |
| 8 | 1.11928 | 1.11980 | 1.18710 | 0.93416 |  |  |  |  |
| 24 | 0.05149 | 0.03599 | 0.05164 | 0.02911 | 0.05665 | 0.11679 | 0.11769 |  |
| 48 | 0.00558 | 0.00634 | 0.00483 | 0.00483 | 0.00558 | 0.04115 | 0.01852 | 0.06789 |
| **HT29 bland bead** | | | | | | | | |
| **Time (hr)** | **Trial 1** | **Trial 2** | **Trial 3** | **Trial 4** | **Trial 5** | **Trial 6** | **Trial 7** | **Trial 8** |
| 1 | 0.98242 | 1.00285 | 1.05606 |  |  |  |  |  |
| 2 | 1.02166 | 1.01545 | 1.01997 |  |  |  |  |  |
| 4 | 1.03779 | 1.00053 | 1.04158 |  |  |  |  |  |
| 8 | 1.13186 | 1.06946 | 1.11316 |  |  |  |  |  |
| 24 | 1.02207 | 0.91174 | 1.06823 | 0.97049 | 1.01163 |  |  |  |
| 48 | 0.95443 | 0.98551 | 0.96937 | 0.95636 | 1.02866 | 0.95489 |  |  |
| **HepG2 drug** | | | | | | | | |
| **Time (hr)** | **Trial 1** | **Trial 2** | **Trial 3** | **Trial 4** | **Trial 5** | **Trial 6** | **Trial 7** | **Trial 8** |
| 1 | 0.42431 | 0.52127 | 0.60663 | 0.71022 | 0.47072 |  |  |  |
| 2 | 0.35198 | 0.52773 | 0.39098 | 0.53415 | 0.48478 |  |  |  |
| 4 | 0.10880 | 0.17971 | 0.08329 | 0.24912 | 0.27126 |  |  |  |
| 8 | 0.00908 | 0.02598 | 0.01972 | 0.09484 | 0.11674 |  |  |  |
| 24 | 0.01008 | 0.00964 | 0.01008 | 0.01052 | 0.01096 | 0.00958 | 0.00919 | 0.00919 |
| 48 | 0.01436 | 0.01299 | 0.01573 | 0.01299 | 0.01368 | 0.00601 | 0.00742 | 0.00813 |
| **HepG2 bland bead** | | | | | | | | |
| **Time (hr)** | **Trial 1** | **Trial 2** | **Trial 3** | **Trial 4** | **Trial 5** | **Trial 6** | **Trial 7** | **Trial 8** |
| 1 | 0.91823 | 0.86602 | 0.87928 |  |  |  |  |  |
| 2 | 0.92858 | 0.90193 | 0.91328 |  |  |  |  |  |
| 4 | 0.93422 | 0.96348 | 0.89557 |  |  |  |  |  |
| 8 | 0.96620 | 0.94085 | 0.98153 |  |  |  |  |  |
| 24 | 1.02981 | 0.94082 | 1.04910 | 1.06270 | 0.80411 | 0.89414 |  |  |
| 48 | 1.32528 | 1.33485 | 1.20903 | 1.52244 | 0.71528 | 1.31712 |  |  |

Measurement data represent the calculated viability (fluorescence signal in bead treated sample / mean fluorescence signal in 3 untreated samples) in relation to an untreated control group for HCT116, HT29, and HepG2 cells exposed to bland and sunitinib DEB over time.
